# P-1067. Navigating the Dual Threat: Clinical Outcomes in Patients with KPC and NDM Co-producing Enterobacterales Infections

**DOI:** 10.1093/ofid/ofaf695.1262

**Published:** 2026-01-11

**Authors:** Howard L Chhen, Tho H Pham, Vanthida Huang

**Affiliations:** Midwestern University College of Pharmacy - Glendale Campus, Glendale, AZ; R. Ken Coit College of Pharamcy/Arizona Department of Health Services, Phoenix, Arizona; Midwestern University, College of Pharmacy-Glendale Campus, Glendale, Arizona

## Abstract

**Background:**

Carbapenemase-producing Enterobacterales (CRE) have continued to rise with high mortality. While *Klebsiella pneumoniae* carbapenemase (KPC) remains the most common carbapenemase, New Delhi metallo-β-lactamases (NDM) have significantly increased. Limited *in vitro* studies have reported CRE isolates co-producing KPC and NDM (KPC+NDM CRE); however, the impact of KPC+NDM CRE on patient outcomes remains unclear.

**Methods:**

This was a multicenter, retrospective cohort conducted at HonorHealth Network (Phoenix, AZ). Adults admitted between September 2022 and March 2025 with KPC+NDM CRE infections were included. The detection and differentiation of carbapenemases were performed with NG-Test CARBA 5 (NG Biotech, Guipry-Messac, France). The primary outcome was clinical cure at discharge. The secondary outcomes were 30-day all-cause mortality, intensive care unit (ICU) and hospital length of stay (LOS).

**Results:**

Overall, 8 patients had confirmed KPC+NDM CRE infections with the mean age of 57 (29-82) years and mean APACHE-II score of 17 (8-30). At baseline, 6 (75.0%) patients required mechanical ventilation, 5 (62.5%) were hospitalized within the past 90 days, and 2 had CRE infection within 90 days prior to admission. The majority of patients (62.5%) were admitted from long term care facilities. *K. pneumoniae* was isolated in 7 (87.5%) patients, while *Proteus mirabilis* was isolated in 1 (12.5%) patient. Clinical cure was achieved in 7 (87.5%) patients with blood and cellulitis as the most common infection sources. Two patients with KPC+NDM CRE bacteremia were successfully managed with ceftazidime/avibactam plus aztreonam and eravacycline, respectively. The repeat blood cultures were clear within 24 hours of therapy initiation. Eravacycline was used for 1 (12.5%) intra-abdominal abscess, 1 (12.5%) cellulitis, and 1 (12.5%) osteomyelitis with the average treatment duration of 23 (7-42) days. The overall mean ICU and hospital LOS were 11(0-29) days and 22 (4-68) days.

**Conclusion:**

In this limited cohort of patients with KPC+NDM CRE infections, a surprisingly high rate of clinical cure was observed. Future larger, prospective studies are warranted to comprehensively characterize the clinical impacts of CRE infections harboring KPC+NDM and other combinations of carbapenemases.

**Disclosures:**

All Authors: No reported disclosures

